# Molecular Responses to Temperature Changes Across Timescales in the Madagascar Ground Gecko (*Paroedura picta*)

**DOI:** 10.1111/mec.70245

**Published:** 2026-01-17

**Authors:** Fuku Sakamoto, Shunsuke Kanamori, Félix Rakotondraparany, Takashi Makino, Masakado Kawata

**Affiliations:** ^1^ Graduate School of Life Sciences Tohoku University Sendai Japan; ^2^ Département Zoologie et Biodiversité Animale Université D'antananarivo Antananarivo Madagascar; ^3^ Institute of Liberal Arts and Sciences Tohoku University Sendai Japan

**Keywords:** ATAC‐seq, ectotherm, phenotypic plasticity, reptile, RNA‐seq, thermal ecology

## Abstract

Short‐term responses to temperature stress, such as heat waves and long‐term acclimation to temperature changes, including seasonal shifts, are expected to be mediated by distinct molecular pathways. However, in ectotherms, such as reptiles, the effects of exposure duration on molecular responses to temperature change remain unclear. In this study, we investigated temperature‐induced molecular changes across distinct timescales in a newly established reptilian model species, the Madagascar ground gecko ( *Paroedura picta* ). To determine temperature‐responsive phenotypes and assess phenotypic plasticity under long‐term temperature changes, we compared thermal traits in individuals acclimated to 25°C and 30°C for more than 30 days. We found significant differences in the critical thermal minimum and maximum as well as sprint speed between the two groups. We then employed RNA sequencing and the assay for transposase‐accessible chromatin using sequencing to analyse gene expression, splicing and chromatin states across multiple temperature conditions and durations. Results revealed that abrupt temperature shifts activated known heat stress pathways, whereas prolonged temperature acclimation altered immune function. In the liver, the predicted occupancy of some transcription factors diverged between short‐ and long‐term temperature stimuli. These findings indicate that transient temperature stress responses and long‐term temperature acclimation in *P. picta* involve distinct molecular mechanisms.

## 1 Introduction

As climate change intensifies, interest is growing in the extent to which organisms can buffer the effects of temperature variation, including heatwaves and the year‐to‐year increase in ambient temperatures, and in how both the capacity and the characteristics of such plasticity influence biodiversity. Ectotherms, such as reptiles, which rely on external heat sources to regulate their body temperature, are particularly vulnerable (Burraco et al. 2020). However, the mechanisms that generate temperature‐induced plasticity in ectotherms, as well as the limits of these responses, remain insufficiently documented.

Differences in the physiological and behavioural responses that allow organisms to adjust to environmental temperature variation arise from genetic factors, plastic responses to environmental change and their interactions (Hendry 2016). Quantifying environmentally induced plasticity in these traits and elucidating its mechanisms are crucial for understanding how organisms cope with temperature variation and predicting selective pressures. Although the importance of plastic responses in the context of climate change has been discussed, and although extinction risks under climate change have been extensively studied, a framework considering plasticity is lacking (Sgrò et al. 2016; Donelson et al. 2023). Identifying the mechanisms underlying plastic responses to environmental change remains a major challenge (Sinclair et al. 2016; Taylor et al. 2021). In reptiles, temperature‐induced phenotypic plasticity has been documented (Domínguez‐Guerrero et al. 2021; Drummond et al. 2020; Rodgers and Franklin 2021; Ryan and Gunderson 2021); however, its direction is inconsistent, with its expression varying depending on factors such as phylogenetic group, temperature level, timescale and environmental interactions. Thus, a mechanistic understanding supported by molecular evidence is needed to improve predictions of reptiles' biological responses to climate change.

Plastic responses to temperature depend on the intensity and duration of exposure. For example, acute exposure to thermal stresses, such as cold or heat waves and long‐term changes, such as seasonal shifts and the overall increase in ambient temperatures over the years, affect organisms differently (Kefford et al. 2022). Although research remains limited, there is evidence in reptiles suggesting that the timescale of thermal exposure can lead to different biological effects (Hall and Sun 2021; Refsnider et al. 2021). The review by Hall and Sun (2021) notes that, at least in embryos, brief exposure to extreme temperatures can result in mortality through immediate physiological failure, whereas prolonged exposure to moderately high temperatures can lead to death following developmental damage or depletion of energy stores. Additionally, at least in fish and mammals, different pathways mediate the responses to short‐ and long‐term thermal exposure (Collier and Gebremedhin 2015; Schulte et al. 2011). Acute heat stimuli trigger cellular‐level heat shock responses and organismal‐level physiological responses, such as altered heart rate, whereas prolonged heat exposure (days to weeks) reprogrammes gene expression and modifies metabolic pathways and homeostatic signalling (Collier and Gebremedhin 2015). Several studies have identified genes involved in reptilian temperature responses (Akashi et al. 2016; Chang et al. 2022; Rosso et al. 2024), but each study focused on arbitrary timescales. To the best of our knowledge, no study has explicitly distinguished the effects of temperature exposure duration in reptiles. Considering timescale in molecular research would provide a higher‐resolution understanding of temperature responses.

A comprehensive understanding of the molecular mechanisms that shape responses to temperature requires not only accumulated knowledge of individual gene expression profiles, but also insight into the upstream regulatory systems that govern them. In open chromatin regions, regulatory elements such as transcription factors can physically interact with DNA sequences, thereby enabling active regulation of gene expression (Klemm et al. 2019). Assay for Transposase‐Accessible Chromatin with high‐throughput sequencing (ATAC‐seq) allows efficient detection of open chromatin regions with relatively small amounts of input material compared with conventional methods (Buenrostro et al. 2013, 2015). Integrating chromatin accessibility profiles obtained by ATAC‐seq with transcriptional data can therefore facilitate the identification of genomic regions and regulatory factors involved in temperature responses. At least in rats, epigenetic mechanisms have been implicated in the transcriptional reprogramming that occurs during thermal acclimation (Horowitz 2016). Although chromatin‐level responses to temperature have not yet been examined in reptiles, studies in other taxa such as rats (Dou et al. 2022), oysters (Wang, Jiang, et al. 2024; Wang, Xie, et al. 2024), and corals (Weizman and Levy 2019) have documented temperature‐related shifts in chromatin accessibility and have identified genomic regions and transcription factors that are likely to contribute to accompanying changes in gene expression. Integrating genome‐wide profiles of gene expression and chromatin accessibility makes it possible to link the roles of individual genes and situate them within a more comprehensive regulatory network, thereby advancing our understanding of the molecular basis that shapes organismal responses to temperature.

The Madagascar ground gecko ( *Paroedura picta* ), a nocturnal species native to Madagascar, has garnered attention as a reptile model owing to its ease of care and year‐round breeding (Noro et al. 2009). This species is distributed in the arid and semi‐arid regions of southern and southwestern Madagascar and is the most terrestrial member of the genus *Paroedura*. It is typically found within leaf litter in dry forest or on sandy substrates in spiny scrub vegetation (Schönecker 2008). Importantly, whole‐genome sequencing has been completed (Hara et al. 2018; Yamaguchi et al. 2021), and genome editing has been reported (Abe et al. 2023). Studies on this species have explored the relationships between embryonic development temperature and posthatching thermoregulatory behaviour (Blumberg et al. 2002), female rearing temperature and egg production (Kubička et al. 2012), and rearing temperature and both body size (StarostovÁ et al. 2010) and cell size (Czarnoleski et al. 2017). However, no studies have quantitatively measured adult thermal response indices, such as critical temperatures, limiting comparisons with other species.

In this study, using *P. picta* , we aim to elucidate how ectothermic reptiles respond differently to acute thermal stress and long‐term temperature changes, particularly at the molecular level. To establish a necessary foundation for interpreting the molecular responses, we first examined whether long‐term thermal acclimation induces plastic changes in physiological and behavioural thermal traits that are consistent with the prevailing environmental temperatures. Based on this assessment, we then investigated the molecular‐level responses underlying such temperature‐dependent plasticity under the framework of the following two hypotheses. First, we hypothesised that long‐term thermal acclimation would elicit molecular responses distinct from those induced by short‐term heat exposure. Specifically, we can predict sustained warm conditions to (i) promote the re‐adjustment of metabolic and other homeostatic processes to the acclimation temperature, and (ii) reduce excessively strong heat‐stress responses that characterise acute exposure. These long‐term regulatory adjustments were predicted to contribute to improved thermal performance at the acclimation temperature, in a manner that is not simply an extension of the acute heat‐stress response. Second, we hypothesised that the temperature‐induced changes in transcriptional profiles are underlain by alterations in chromatin structure and transcription factor binding, which may respond in different ways across acute heat exposure and long‐term thermal acclimation. By simultaneously examining the effects of temperature across multiple molecular layers, including chromatin accessibility, gene expression and splicing, we can test these two hypotheses and clarify how different aspects of the molecular response in *P. picta* are interconnected and thereby improve our understanding of the molecular regulatory networks that operate in response to temperature.

To address these hypotheses, we first identified the phenotypic plasticity of this species in response to long‐term environmental temperature change, and then characterised chromatin accessibility and transcriptomic variation under thermal treatments that differed in duration. We measured four widely used thermal response indices (Taylor et al. 2021): selected temperature (T_sel_), that is, the body temperature an individual chooses; critical thermal minimum (CT_min_) and critical thermal maximum (CT_max_), representing the lower and upper physical activity limits, respectively; and performance (in this study, sprint speed) across various body temperatures. Using these measurements, we estimated the thermal performance curve (TPC) for locomotion, describing the relationship between body temperature and performance. To determine whether long‐term temperature changes induce phenotypic changes, we compared thermal response indices in geckos reared at different temperatures, confirming that traits adjust plastically to environmental temperature. Finally, we explored gene transcription and chromatin structure changes in response to short‐ and long‐term temperature shifts using RNA sequencing (RNA‐seq) and the assay for transposase‐accessible chromatin using sequencing (ATAC‐seq). Together, these analyses describe the temperature‐dependent responses occurring at these distinct levels and provide a foundation for future work aimed at elucidating their functional relationships.

## 2 Materials and Methods

### 2.1 Habitat Temperature of *P. picta*

To determine appropriate rearing temperatures for *P. picta* , we analysed daily air temperature data (average, minimum and maximum) during 2010–2020 for three cities in Madagascar (Morondava, Taolagnaro and Toliara) located near its habitat, as previously described by Schönecker (2008). Data were sourced from the National Oceanic and Atmospheric Administration database (https://www.ncei.noaa.gov/cdo‐web/). Daily mean temperatures were calculated by averaging the available daily mean temperature values for each calendar day across years (2010–2020). The average daily mean temperature across all days of the year was 24.0°C–26.5°C across the three cities, whereas the highest daily mean temperature of the year was 27.9°C–32.7°C (Figure S1 and Table S1). Based on these data, two rearing temperatures were selected: 25°C ± 1°C, representing the annual average habitat temperature and 30°C ± 1°C, approximating the average daily temperature during the hottest period of the year.

### 2.2 Animal Preparation and Experimental Design

Animals were obtained from a licenced laboratory supplier (Meito Suien, Aichi, Japan). All care and experiments followed the guidelines of the Animal Experimentation Committee of Tohoku University, Sendai, Japan (approval no.: 2020LsA‐008‐03). Use of *P. picta* genetic information was approved by Madagascar's General Management of Environmental Governance (no. 176/23/MEDD/SG/DGGE/DAPRNE/SCBE.Re).

Sexually mature adults were individually housed under controlled environmental conditions (relative humidity > 45%; 12:12‐h light:dark cycle, with light from 00:00 to 12:00). Cages provided water and shelter, and crickets dusted with nutritional supplements were given as food every 2–3 days. Animals were not fed for at least 12 h before experiments.

**FIGURE 1:**
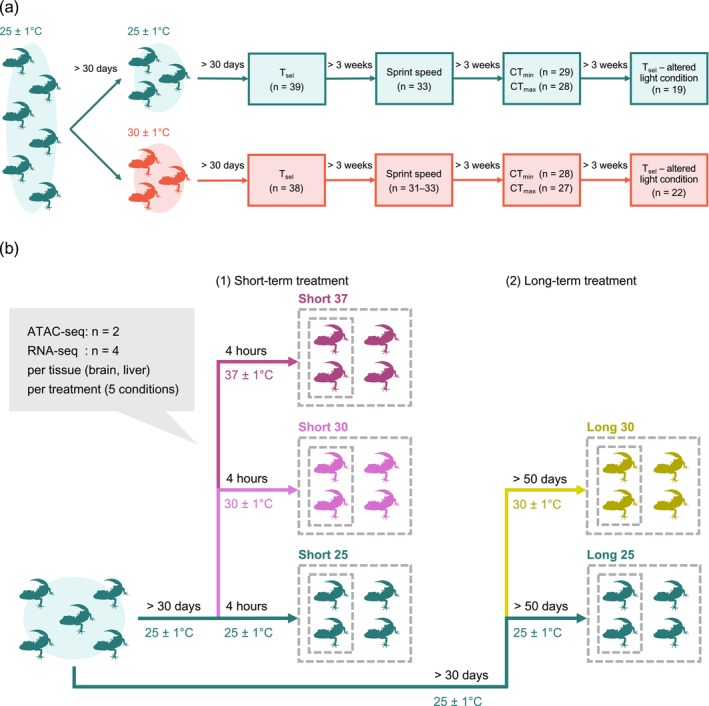
(a) Experimental design and sample sizes for behavioural experiments. All individuals were first maintained at 25°C ± 1°C for more than 30 days. They were then acclimated for more than 30 days at either 25°C ± 1°C or 30°C ± 1°C. Assays were conducted in the following fixed order: (1) selected temperature (T_sel_) under the standard light condition, (2) sprint speed, (3) critical thermal limits (critical thermal minimum, CT_min_; and critical thermal maximum, CT_max_) and (4) T_sel_ under an altered light condition. More than 3 weeks were allowed between each assay. CT_min_ and CT_max_ were measured in counterbalanced order, with more than 3 weeks between the two measurements. Sample sizes for sprint speed are shown as ranges because the number of individuals that yielded reliable measurements varied slightly among tested temperatures. In the 25°C acclimation group, five individuals were additionally measured for T_sel_ under the altered light condition immediately after the acclimation period in order to increase the sample size for this assay. (b) Experimental design for RNA‐seq and ATAC‐seq. All individuals were first acclimated at 25°C ± 1°C for over 30 days, after which samples were collected following five temperature treatments (Short 25, Short 30, Short 37, Long 25 and Long 30) as illustrated in the figure. Short‐term treatments involved a 4‐h exposure to 25°C, 30°C or 37°C, whereas long‐term treatments consisted of over 50 days of exposure to either 25°C or 30°C. RNA‐seq was used to assess temperature‐dependent changes in gene expression and splicing, and ATAC‐seq was used to characterise associated shifts in chromatin accessibility and transcription factor footprints. For each treatment, brain and liver were sampled (RNA‐seq: *N* = 4 per tissue; ATAC‐seq: *N* = 2 per tissue).

For behavioural experiments, T_sel_, CT_min_, CT_max_ and sprint speed at various body temperatures were measured to assess temperature response phenotypes. The sequence of assays followed the overall order presented in Figure 1a: initial measurement of T_sel_ under the standard light condition, followed by sprint speed trials, assessments of critical thermal limits (CT_min_ and CT_max_) and a final T_sel_ measurement under an altered light condition. Experiments were performed more than three weeks apart. CT_min_ and CT_max_ were administered in counterbalanced order across individuals, with more than three weeks between the two measurements. To test for plastic changes, individuals were housed at 25°C ± 1°C for more than 30 days, followed by housing at 25°C ± 1°C or 30°C ± 1°C for more than 30 days before measurement. Body weight was recorded before experiments, and snout–vent length was measured within one week after experiments. In the 25°C acclimation group, several individuals were additionally measured for T_sel_ under the altered light condition immediately after the acclimation period to increase the sample size for that assay.

RNA‐seq and ATAC‐seq were performed using samples collected after different temperature treatments to distinguish molecular responses to varying thermal stimuli (Figure 1b). Individuals were first acclimated at 25°C ± 1°C for at least 30 days before being subjected to the following treatments: for short‐term treatments, individuals were exposed to 25°C (approximating the annual average habitat temperature), 30°C (approximating the daily average habitat temperature during the warmest period) or 37°C (approximating the maximum habitat temperature) for 4 h (referred to as Short 25, Short 30 and Short 37, respectively); for long‐term treatments, individuals were exposed to 25°C or 30°C for > 50 days (referred to as Long 25 and Long 30, respectively). RNA‐seq was used to detect changes in gene expression and splicing in the short‐term (30°C and 37°C) and long‐term (30°C) treatment groups, compared to the 25°C control. Similarly, ATAC‐seq was applied to identify changes in chromatin accessibility and transcription factor footprints between the treatment groups and the control. For the short‐term treatments, individuals were placed in an incubator at the target temperature (±1°C) for 4 h with free access to water. Long‐term treatments followed standard housing conditions, except for the incubator temperature.

All individuals used were adult males, and both brain and liver samples were collected. RNA‐seq included 40 samples from 20 individuals, with 4 samples per temperature condition and tissue type. ATAC‐seq included 20 samples from 10 individuals, with 2 samples per condition and tissue type. After temperature treatment, animals were immediately decapitated, and tissues were either processed for ATAC‐seq or snap‐frozen in liquid nitrogen and stored at −80°C.

### 2.3 T_sel_ Measurement

Given that thermoregulatory behaviours, such as basking, depend on the habitat's light environment, measurements were taken under both light and dark conditions. An aluminium plate (50 × 70 cm) covered with black cloth had one long‐side end cooled with ice packs (replaced every 2 h) and the other end heated with a far‐infrared heater, creating a temperature gradient from 10°C–15°C to 42°C–47°C. Individuals were placed in a track (6 × 70 cm) on the plate, allowing free movement and cloacal temperature was recorded every 2 h for 6 total measurements under each condition. The first measurement was taken 1 h after the individual was introduced into the track to allow habituation. The average measurement for each condition was taken as T_sel_ for that condition. Light and dark measurements occurred on separate days, with randomised condition order by groups of several individuals. To isolate light effects from time of day, measurements were also taken under dark conditions during the light period and light conditions during the dark period. The Wilcoxon signed‐rank test was used for paired data to assess differences in T_sel_ between light and dark conditions, whereas the Wilcoxon rank‐sum test was used for unpaired data to compare acclimation temperatures.

### 2.4 CT_min_ and CT_max_ Measurements

For CT_min_, following previous studies that induced cooling using ice or ice packs (e.g., Gilbert and Miles 2016; Romero‐Báez et al. 2020), individuals were placed on an ice pack, and their body temperature was recorded every minute. Once body temperature dropped below 16°C (for 25°C‐acclimated individuals) or 20°C (for 30°C‐acclimated individuals), each individual's righting response was tested every minute by placing them on their back. The temperature at which the individual could no longer right itself within 30 s, even with forceps stimulation, was recorded as CT_min_.

For CT_max_, following earlier work that used external heat sources to elevate body temperature (e.g., Gilbert and Miles 2016; Romero‐Báez et al. 2020), individuals were placed in an incubator at 55°C–58°C, and their body temperature was recorded every minute, taking caution to avoid rapid increases in the temperature that could cause significant discomfort. Once this temperature exceeded 33°C, their righting response was tested as described above, with CT_max_ recorded as the temperature at which self‐righting failed within 30 s.

The experiments targeted a body temperature change rate of approximately 1°C/min (observed mean rates: CT_min_, 1.23°C/min [0.82–2.33°C/min]; CT_max_, 1.27°C/min [0.67–1.72°C/min]). Differences between acclimation groups were evaluated using permutation ANCOVA (50,000 permutations; seed = 123) with body temperature change rate included as a covariate.

### 2.5 Measurement of Sprint Speed

Sprint speed was measured at 15°C, 20°C, 25°C, 30°C, 35°C and 37°C. Before trials, individuals were placed in an incubator for 1.5 h to reach the target body temperature. Body temperature was measured before and after runs to confirm that it remained within ±1°C of the target. If an individual stopped moving for over 1 min or > 5 min had elapsed since the trial began, recorded data up to that point were used. Each temperature was tested three times, with at least 30 min between trials. Trials were conducted over 6 days, with a different temperature tested daily. The order of test temperatures was randomised for 1–2 individuals. As *P. picta* is nocturnal, all measurements were performed in darkness (illuminance < 0.2 lx) during the dark period. Each individual with a phosphorescent sticker attached to their back was placed at one end of a track (6 × 110 cm), and escape behaviour was induced by stimulating near the hindlimbs with a brush. A video camera (HC‐V480MS‐W, Panasonic, Osaka, Japan) was used to record trials, and Kinovea v0.9.5 (www.kinovea.org) was employed to track movement via the phosphorescent sticker. Speed was calculated every 0.1 s, with the highest speed from three trials recorded as sprint speed at that body temperature.

Sprint speed was analysed using a permutation‐based repeated‐measures ANOVA for a split‐plot design (50,000 permutations; seed = 123), with acclimation temperature as a between‐subject factor and test temperature as a within‐subject factor. When a significant interaction was detected, simple main effects of acclimation temperature were evaluated at each test temperature using permutation tests, and the resulting *p*‐values were adjusted using the Holm method.

### 2.6 TPC Estimation

TPCs, describing the relationship between body temperature and physical performance, were modelled using CT_min_, CT_max_ and sprint speed data, with CT_min_ and CT_max_ set as zero performance points. The model followed Kamykowski (1985), with predicted performance constrained to non‐negative values. The initial value, lower limit and upper limit of each model parameter were estimated using rTPC v1.0.4 (Padfield et al. 2021), an R package for nonlinear least‐squares TPC estimation. Model parameters were then estimated via hierarchical Bayesian inference using cmdstanr v0.8.0 (4 chains; 1000 warmup + 1000 sampling iterations per chain; adapt_delta = 0.95). The hierarchical model included population‐level parameters and individual‐level deviations, and logit reparameterisation together with non‐centred parameterisation was used to improve sampling efficiency and enforce parameter bounds. For each posterior draw, maximum performance (P_max_) and the corresponding optimal body temperature (T_opt_) were numerically determined using a two‐stage grid search (50‐point coarse grid followed by 100‐point fine grid), yielding posterior distributions for these derived variables. TPCs were estimated separately for individuals acclimated to 25°C and 30°C. The scripts used for TPC estimation are available on Dryad (https://doi.org/10.5061/dryad.pvmcvdnwm).

### 2.7 RNA‐Seq and Transcriptome Analysis

Total RNA was extracted using the Maxwell RSC Plant RNA Kit and Maxwell RSC Instrument AS4500 (Promega, Madison, WI, USA). RNA concentration and purity were verified using a Nanodrop spectrophotometer (NanoDrop Technologies LLC, Wilmington, DE, USA) and an Agilent Bioanalyzer (Agilent Technologies Inc., Santa Clara, CA, USA). Library preparation and sequencing were outsourced to Novogene (Pledran, France) via Filgen Inc. (Aichi, Japan), with library sequencing performed on a NovaSeq6000 system (Illumina Inc., San Diego, CA, USA) under PE150 conditions.

Reads were quality‐checked using fastp v0.23 (Chen et al. 2018) and mapped to the reference genome (Hara et al. 2018) using STAR v2.7.11 (Dobin et al. 2013). Gene expression levels were quantified using RSEM v1.3.1 (Li and Dewey 2011). Principal component analysis (PCA) was performed on gene transcripts per kilobase million values using the ‘prcomp’ function in R v4.2.2 (http://www. R‐project.org/). Differentially expressed genes (DEGs) between temperature conditions were identified via Wald tests in DESeq2 v1.38.3 (Love et al. 2014), with a false discovery rate (FDR) of < 0.05 following *p*‐value correction using the Benjamini–Hochberg method. Differentially spliced genes (DSGs) were detected using rMATS v4.3.0 (Wang, Jiang, et al. 2024; Wang, Xie, et al. 2024), with the ‘—novelSS’ option applied to predict novel splice site, as the genome annotation lacked isoform information. DSG detection criteria followed Wang, Jiang, et al. (2024); Wang, Xie, et al. (2024). Gene names and Gene Ontology (GO) annotations were linked to gene IDs using EnTAP v1.0.1 (Hart et al. 2020).

Weighted gene co‐expression network analysis (WGCNA; Langfelder and Horvath 2008) was performed on RNA‐seq data using the WGCNA R package (v1.73). Gene expression was filtered (CPM > 1 in ≥ 20% of samples), normalised using the variance‐stabilising transformation (DESeq2) and the top 5000 most variable genes were retained. Sample outliers were identified and removed by hierarchical clustering with average linkage (height cutoff = 150). The soft‐thresholding power was selected as the lowest power achieving a scale‐free topology fit *R* ^2^ > 0.80 based on Pearson correlation. A signed co‐expression network was then constructed using biweight midcorrelation with the selected power. Modules were identified using blockwiseModules (deepSplit = 2, minimum module size = 30, merge cut height = 0.25). Module‐trait associations were quantified by computing biweight midcorrelation between module eigengenes and experimental groups, which were encoded as binary indicator variables. *p*‐values were obtained using the Student asymptotic approximation and adjusted for multiple testing using the Benjamini–Hochberg procedure (FDR < 0.05). Hub genes were defined as genes with absolute module membership (|kME|) > 0.8 in modules significantly associated with traits (FDR < 0.05). As an overall measure of network stability, we performed 50 bootstrap resamplings of samples with replacement, recalculated module eigengenes and kME values while retaining original module assignments, and for each bootstrap iteration computed the mean kME across genes and modules; the network stability score was defined as the average of these mean kME values. The script used for the WGCNA analysis is available at Dryad (https://doi.org/10.5061/dryad.pvmcvdnwm).

GO enrichment analysis of DEGs, DSGs and genes assigned to each WGCNA module was performed using the R package goseq v1.50.0 (use_genes_without_cat = TRUE, method = ‘Wallenius’; Young et al. 2010). GO terms were identified as enriched at FDR < 0.05, with multiple testing correction achieved using the Benjamini–Hochberg method, considering all annotated genes as the background.

### 2.8 ATAC‐Seq and Chromatin Accessibility Analysis

The ATAC‐seq library was prepared using the ATAC‐seq Kit (Active Motif, Carlsbad, CA, USA) following the kit protocol with modifications. Specifically, during nuclear extraction, fragmented tissue was washed up to three times with phosphate‐buffered saline to remove contaminants. DNA fragments underwent 10–12 polymerase chain reaction cycles, followed by double‐size selection of the DNA library using Solid Phase Reversible Immobilisation select beads (Beckman Coulter, IN, USA) to remove extremely small or large fragments.

Sequencing was outsourced to BGI Genomics (Wuhan, China) and performed using the DNBSEQ G‐400 platform (MGI Tech Co. Ltd., Shenzhen, China) under PE100 conditions. Read quality control, mapping, filtering and peak calling were conducted using the nf‐core/atac‐seq pipeline (Ewels et al. 2020; Patel et al. 2023) with default parameters, except peak calling being set to ‘narrow peak’ mode, and mitochondrial reads were removed using the ‘mito_name’ command. In this filtering, BLASTN 2.14.0+ (Zhang et al. 2000) was employed to identify homology between the species' entire genome and its complete mitochondrial sequence (Starostová and Musilová 2016), pinpointing ‘scaffold00001683’ as the predicted mitochondrial sequence.

Chromatin accessibility differences between temperature conditions were analysed using the edgeR method in DiffBind v3.4.11 (Stark and Brown 2012), with significant regions detected at FDR < 0.05 following multiple testing correction of *p*‐values using the Benjamini–Hochberg method. Transcription factor footprint scores were calculated using TOBIAS v0.16.1 (Bentsen et al. 2020), with a nonredundant set of vertebrate transcription factor binding motifs obtained from JASPAR CORE 2024 (Rauluseviciute et al. 2023). Gene‐proximal regions (within 5‐kb upstream/downstream of genes or 1000‐bp upstream to 100‐bp downstream of gene start sites) were extracted using the ‘slop’ and ‘flank’ commands of bedtools v2.30.0 (Quinlan and Hall 2010), with the peaks overlapping gene‐proximal regions identified using the ‘intersect’ command.

## 3 Results

### 3.1 Plasticity of Thermal Traits in Response to Long‐Term Temperature Acclimation

To assess thermal response in *P. picta* , we measured T_sel_, CT_min_, CT_max_ and sprint speed across various body temperatures. Additionally, we examined differences in these thermal traits after acclimation to 25°C and 30°C to evaluate long‐term thermal plasticity.

**FIGURE 2:**
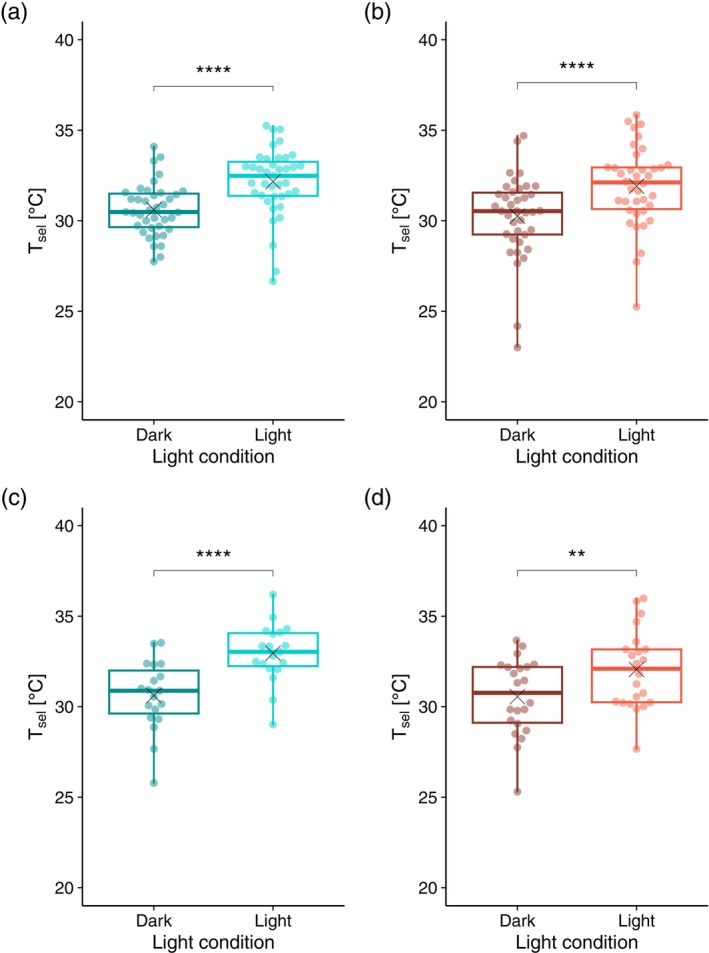
Selected temperature (T_sel_) under different light conditions. (a) 25°C acclimation; (b) 30°C acclimation; (c) 25°C acclimation under dark and light conditions with the time of day set to ‘light’ and ‘dark’, respectively; and (d) 30°C acclimation under dark and light conditions with the time of day set to ‘light’ and ‘dark’, respectively. Each data point represents an individual measurement. **** and ** indicate significant differences between light conditions based on the Wilcoxon signed‐rank test, with *p* < 0.0001 and *p* < 0.01, respectively.

T_sel_ did not differ significantly between 25°C‐ and 30°C‐acclimated individuals under either light or dark conditions. However, both groups exhibited significantly higher T_sel_ under light conditions than under dark conditions (25°C: *p* = 4.40 × 10^−6^; 30°C: *p* = 8.09 × 10^−7^; both Wilcoxon signed‐rank tests; Figure 2a,b; Table S2). To differentiate between brightness and time‐of‐day effects, T_sel_ was measured under light conditions during the dark period (12:00–00:00) and under dark conditions during the light period (00:00–12:00) in the rearing cycle. In both groups, T_sel_ remained significantly higher under light conditions (25°C: *p* = 1.14 × 10^−5^; 30°C: *p* = 0.00251; both Wilcoxon signed‐rank tests; Figure 2c,d; Table S2), with values under light conditions during the dark period being more closely aligned with those measured under light conditions during the light period.

**FIGURE 3:**
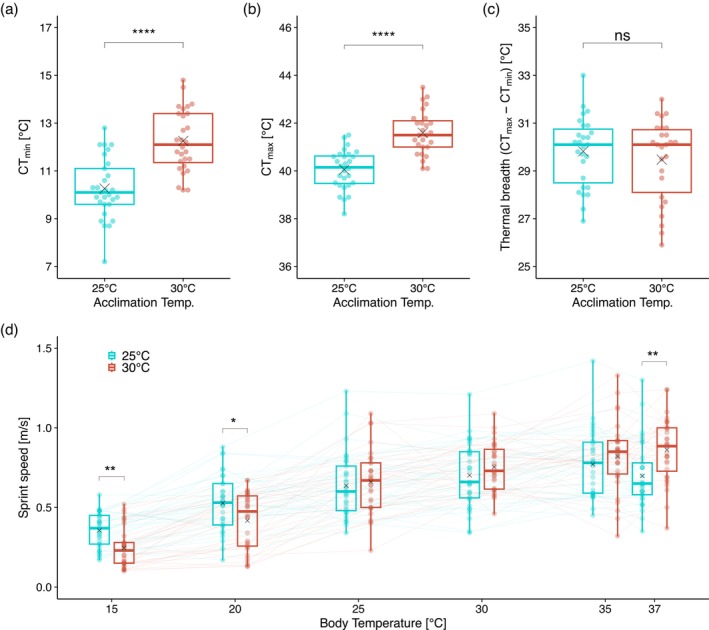
Critical thermal minimum (a: CT_min_), critical thermal maximum (b: CT_max_), thermal breadth (c: CT_max_−CT_min_) and sprint speed at various body temperatures (d). In (a–c), significance between acclimation temperatures was tested using permutation ANCOVA (****p < 0.0001; ns *p* > 0.05). In (d), differences between acclimation temperatures at each test temperature were assessed by post hoc permutation tests following a split‐plot permutation ANOVA. *p*‐values were adjusted using the Holm method (*p < 0.05; **p < 0.01).

CT_min_ (Figure 3a and Table S3) and CT_max_ (Figure 3b and Table S3) were significantly lower in 25°C‐acclimated individuals (CT_min_: F_1,54_ = 46.08, *p* < 2 × 10^−5^; CT_max_: F_1,52_ = 45.05, *p* < 2 × 10^−5^; both permutation ANCOVA). However, thermal breadth (CT_max_ − CT_min_) did not significantly differ between the acclimation groups (F_1,49_ = 0.57, *p* = 4.56 × 10^−1^; permutation ANCOVA), indicating a shift in the temperature range at which individuals are active rather than an expansion or contraction of this range (Figure 3c and Table S3).

The effects of body and acclimation temperatures on sprint speed were analysed via a permutation ANOVA for a split‐plot design (Figure 3d and Table S4). Significant effects on sprint speed were detected for test body temperature (F_5,310_ = 141.95, *p* < 2 × 10^−5^) and the interaction between test body temperature and acclimation temperature (F_5,310_ = 10.54, *p* < 2 × 10^−5^). Post hoc test of simple main effects (with Holm's correction) revealed significant differences between acclimation temperatures at 15°C (*p* = 7.1 × 10^−3^), 20°C (*p* = 4.58 × 10^−2^), and 37°C (*p* = 9.1 × 10^−3^).

**FIGURE 4:**
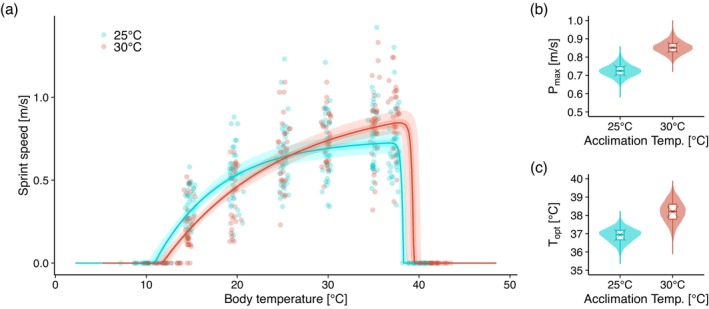
Predictive distributions of the estimated thermal performance curve (a: TPC), distributions of maximum performance (b: P_max_) and distributions of optimal temperature (c: T_opt_) derived from the estimated TPC. The solid line represents the median, with the darker band showing the 50% confidence interval and the lighter band representing the 95% confidence interval. The plots display measured values of critical thermal minimum (CT_min_), critical thermal maximum (CT_max_) or sprint speed for each individual, with sprint speed set to zero for CT_min_ and CT_max_.

TPC estimation using CT_min_, CT_max_ and sprint speed data (Figure 4a) confirmed that 25°C‐acclimated individuals performed better at lower temperatures, whereas 30°C‐acclimated individuals excelled at higher temperatures. The distributions of P_max_ and T_opt_ differed between the acclimation groups, with higher average values in 30°C‐acclimated individuals (P_max_ in 25°C‐acclimated group: 0.73 m/s; P_max_ in 30°C‐acclimated group: 0.85 m/s; T_opt_ in 25°C‐acclimated group: 36.9°C; T_opt_ in 30°C‐acclimated group: 38.2°C; Figure 4b,c).

### 3.2 General Characteristics of Sequencing Datasets

**FIGURE 5:**
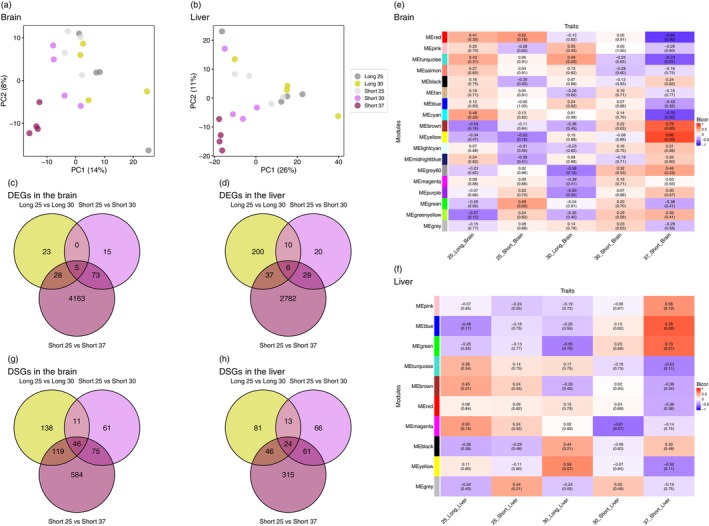
Principal component analysis of gene expression in the brain (a) and liver (b). Each point represents the gene expression profile of an individual sample. Numbers in parentheses indicate the proportion of variance explained by the principal components. PC1 and PC2: First and second principal components, respectively. Numbers of differentially expressed genes (DEGs) in the brain (c) and liver (d). DEGs were identified by comparing each temperature treatment (Long 30: Exposure to 30°C for > 50 days; Short 30 and Short 37: Exposure to 30°C or 37°C for 4 h) to the control group (25°C) over the same duration (Long 25: Exposure to 25°C for > 50 days; Short 25: Exposure to 25°C for 4 h). Venn diagrams indicate the overlap among the three contrasts. Module–trait correlations identified by weighted gene co‐expression network analysis (WGCNA) in the brain (e) and liver (f). Colours represent the strength and direction of the bicor correlation between module eigengenes and experimental groups. Numbers in each cell indicate the correlation coefficient, with the corresponding FDR‐adjusted *p*‐value shown in parentheses. Numbers of differentially spliced genes (DSGs) in the brain (g) and liver (h). DSGs were identified using the same pairwise comparisons as for DEGs. Venn diagrams indicate the overlap among the three contrasts. In (c, d, g, h), yellow, pink and red correspond to Long 25 versus Long 30, Short 25 versus Short 30 and Short 25 versus Short 37, respectively.

To determine whether short‐term (Short 25, Short 30 and Short 37) and long‐term (Long 25 and Long 30) temperature treatments induce distinct molecular responses, we analysed gene transcription and chromatin state using RNA‐seq and ATAC‐seq. RNA‐seq generated 85,638,012–119,565,010 raw reads per sample, with 75.5%–88.2% of quality‐controlled reads mapping to the reference genome (Table S5). PCA revealed distinct clustering of Short 37 and Short 30 samples in both liver and brain tissues (Figure 5a,b and Figure S2), whereas Long 30 samples overlapped with the 25°C treatment samples in at least the first two principal components. These results suggest that short‐term temperature changes induced pronounced transcriptional shifts relative to the 25°C control, whereas long‐term temperature acclimation produced subtler overall expression differences, indicating that gene expression characteristics differ between short‐term and long‐term treatments.

ATAC‐seq generated 73,982,368–273,225,138 raw reads per sample, with 52.9%–69.0% aligning to the genome (Table S6). The number of detected peaks ranged from 62,558 to 213,767. Chromatin accessibility signals were enriched around transcription start sites in all samples, reproducing the general trend observed in ATAC‐seq (Figure S3). PCA of read counts per peak revealed that, in brain samples, Short 25, Short 30 and Short 37 each formed a cluster, whereas Long 30 overlapped with Long 25 (Figure S4). In liver samples, all treatment groups formed distinct clusters without overlap. ATAC‐seq data also indicated distinct effects of short‐term and long‐term temperature treatment, mirroring transcriptomic trends.

### 3.3 DEGs in Response to Short‐ and Long‐Term Temperature Changes

DEGs for each temperature treatment relative to 25°C are listed in Table S7. In the brain, the highest number of DEGs was observed in Short 37, followed by Short 30 and Long 30 (Table S7a). More than half of the genes detected in Short 30 and Long 30 overlapped with those in Short 37 (Figure 5c). In the liver, DEG counts followed a different trend: Short 37 had the most, followed by Long 30 and Short 30 (Table S7b and Figure 5d), with over half of Long 30 DEGs being unique to that treatment.

GO analysis (Table S8) revealed significant enrichment only for Short 37 in the brain, with ‘gene expression’ and ‘mRNA metabolic process’ as the most enriched terms. In the liver, significant enrichment was found in Short 30, Short 37 and Long 30. Shared GO terms between Short 37 and Long 30 included ‘immune system process’ and ‘response to stress’. The only enriched term in Short 30 was ‘defence response to virus’, which was also present in Long 30. Short 37–specific GO terms included ‘protein refolding’, ‘protein ubiquitination’, ‘regulation of autophagy’, ‘entrainment of circadian clock’, ‘response to heat’, and ‘lipid homeostasis’. Long 30 uniquely featured many immune‐related terms, such as ‘innate immune response’, with a high concentration of immune‐associated terms among the top‐ranked results.

### 3.4 Co‐Expression Modules in Response to Short‐ and Long‐Term Temperature Changes

Weighted gene co‐expression network analysis (WGCNA) identified multiple co‐expression modules associated with thermal treatments in liver and brain tissues. Gene lists for all modules detected by WGCNA are provided in Table S9. For modules significantly associated with temperature treatments, GO enrichment results and hub gene lists (kME > 0.8) are summarised in Tables S10 and S11, respectively. In liver, two modules showed significant positive correlations with the 37°C short‐term acclimation group: the blue module (743 genes, *r* = 0.78, FDR = 2.21 × 10^−3^) and the green module (428 genes, r = 0.73, FDR = 6.19 × 10^−3^). In brain, five modules were significantly associated with the same treatment, including two positively correlated modules—yellow (380 genes, *r* = 0.86, FDR = 1.20 × 10^−4^) and brown (723 genes, *r* = 0.78, FDR = 1.53 × 10^−3^)—and three negatively correlated modules—red (326 genes, *r* = −0.84, FDR = 1.41 × 10^−4^), turquoise (894 genes, *r* = −0.73, FDR = 5.33 × 10^−3^) and cyan (49 genes, *r* = −0.76, FDR = 1.93 × 10^−3^). In the liver, the green module also exhibited a moderate negative correlation with the 30°C long‐term acclimation group (*r* = −0.55, *p* = 0.012), although this association was not statistically significant after FDR correction (FDR = 0.104). GO enrichment analysis showed that the liver green module was not only enriched for protein quality control–related processes, such as protein refolding and responses to misfolded or unfolded proteins, but also for broader regulatory and metabolic pathways. These included multiple terms associated with transcriptional regulation, RNA metabolic processes and cellular stress responses, as well as amino acid and lipid metabolic processes (Table S10). Hub genes (kME > 0.8) included several heat shock proteins and chaperones (*HSP90AB1*, *DNAJA4*, *HSPA4L*, *DNAJB6*), transcriptional regulators (*YY1*, *JUND*, *NR4A2*), components of stress‐activated signalling pathways (*DUSP1*, *DUSP16*) and metabolic enzymes such as *GCLC*, *ACLY* and *ASNS* (Table S11). The liver green module shared 53 genes (12.4%) with the brain yellow module. This overlap was significantly greater than expected by chance (Fisher's exact test OR = 3.09, *p* = 1.03 × 10^−10^). The brain yellow module, despite this shared gene membership, did not show a notable association with the 30°C long‐term acclimation group (*r* = 0.096, FDR = 0.880).

### 3.5 DSGs in Response to Short‐ and Long‐Term Temperature Changes

To assess temperature‐induced splicing changes, we identified DSGs in the brain and liver (Table S12a,b, respectively). In both tissues, the highest number of DSGs was found in Short 37, and over half of the DSGs in Short 30 and Long 30 overlapped with those in Short 37 (Figure 5g,h). GO enrichment analysis showed significant enrichment for DSGs in Short 37 and Long 30 in both tissues (Table S13). Across all groups where enrichment was detected, ‘mRNA processing’ and ‘RNA splicing’ were consistently observed. In the brain, additional GO terms included ‘synaptic vesicle budding from presynaptic endocytic zone membrane’ (Long 30) as well as ‘generation of neurons’ and ‘stress granule assembly’ (Short 37). In the liver, Long 30 showed enrichment for ‘tyrosine catabolic process’, ‘translation’, ‘intercellular transport’, and ‘nuclear export’, whereas Short 37 had only one additional enriched term, ‘cellular metabolic process’.

### 3.6 Chromatin Accessibility Changes in Response to Temperature

To examine noncoding region contributions to temperature responses, we analysed chromatin accessibility changes for each temperature condition. The number of significantly differentially accessible peaks in each treatment group relative to 25°C is summarised in Tables S14 and S15. In Long 25, 98,168 peaks showed significant accessibility differences between the brain and liver, whereas the number of peaks with significant accessibility differences across temperature conditions within the same tissue was approximately three orders of magnitude lower. No significant accessibility changes were detected in the liver under Long 30.

Genes with peaks showing accessibility changes within 5‐kb of their upstream or downstream regions were classified as differential peak‐associated genes (DPGs). DPGs overlapping with DEGs or DSGs were identified in each temperature condition, including *HSPA8* (Short 37 in the brain), *PICALM* (Short 30 in the liver), *RRBP1* and *DNAJA4* (both Short 37 in the liver) (Figure S5). Although most DPGs did not overlap with DEGs or DSGs, 85.7%–100% of DEGs and DSGs contained at least one accessibility signal peak within 5‐kb upstream or downstream of the gene under either control or treatment conditions (Table S16).

### 3.7 Changes in Transcription Factor Footprints due to Temperature Exposure

To identify transcription factors involved in temperature‐responsive gene regulation, we analysed footprint score changes, which indicate changes in transcription factor binding site occupancy across temperature treatments relative to 25°C (Table S17a and Figure S6). In the liver, most transcription factors detected in Short 30, Short 37 and Long 30 exhibited opposite occupancy changes between short‐ and long‐term treatments (Table S17b). Among these, CEBPA, CEBPB, CEBPD, CEBPE and CEBPG belong to the C/EBP family, which has been reported to exhibit temperature‐responsive expression changes in other organisms (Bloomer et al. 2014; Buckley 2011; Dou et al. 2022; Sleadd and Buckley 2013). We filtered DEGs with an absolute fold change > 2 in footprint scores for C/EBP family transcription factor binding sites within predicted promoter regions (from 1000‐bp upstream to 100‐bp downstream of the gene start site). Several common DEGs were detected across multiple family members, and a total of 20 DEGs were identified (Table S18).

## 4 Discussion

For ectotherms, such as reptiles, which depend on external heat sources for thermoregulation, climate change poses major challenges (Burraco et al. 2020). Understanding their phenotypic and molecular responses to temperature changes is crucial. Acute heat stress (e.g., from heatwaves) and prolonged warming are expected to elicit distinct biological responses. We sought to investigate the molecular processes that underlie temperature‐induced transcriptional changes across different timescales. To this end, we proposed two hypotheses: (1) distinct molecular responses to short‐ versus long‐term temperature changes and (2) chromatin‐ and transcription factor–based regulation of temperature‐induced transcriptional changes. To test these hypotheses, we first characterised the temperature‐response profile of *P. picta* , an emerging reptilian model species. Our results demonstrated that *P. picta* acclimates to its thermal environment and exhibits plasticity in its temperature‐response phenotype. We then evaluated the hypotheses using RNA‐seq and ATAC‐seq analyses across different temperatures and exposure durations. These molecular analyses revealed distinct transcriptional and regulatory responses depending on the duration of thermal exposure, supporting our hypotheses.

Age information for the individuals used in this study was not available, and the animals included in our assays likely represented a mixture of age classes. Because temperature can influence ageing‐related processes (Burraco et al. 2020), some of the variation observed in our phenotypic and molecular data may reflect age‐related differences that could not be controlled for. Although the interplay between temperature and ageing is an interesting topic in thermal biology, the constraints of our experimental design do not allow us to address it further here.

A separate limitation concerns the potential influence of experimental history within the study. Because the same individuals were used across multiple assays, prior measurements may have had some lingering effects on subsequent traits, even though we incorporated an approximately 3‐week interval between experiments to mitigate such carryover effects. Ideally, each assay would be performed on an independent set of individuals, but the number of animals available was limited. As a result, we cannot fully exclude the possibility that responses observed in later assays were shaped, at least in part, by earlier experimental experiences. We have explicitly acknowledged this limitation in discussing the interpretation of our results.

### 4.1 Light Intensity–Dependent Changes in T_sel_

T_sel_ in *P. picta* was significantly higher under light conditions than under dark conditions, regardless of the time of day, suggesting that this species adjusts its thermoregulation based on light intensity. A similar pattern has been reported in the nocturnal Tokay gecko ( *Gekko gecko* ), which also selects higher body temperatures during light periods (Refinetti and Susalka 1997). In the nocturnal gecko *Amalosia lesueurii*, sprint speed is highly temperature‐dependent during the day, whereas at night, performance remains more stable across temperatures (Dayananda et al. 2020). For *P. picta* , selecting higher body temperatures during inactive periods during the day may help maintain high performance, and this behavioural shift may be modulated by the surrounding light intensity. This could be beneficial, for example, for rapid predator evasion if disturbed while resting. Another possibility is that maintaining lower temperatures during the active phase at night may help offset secondary heat generation by muscle activity or metabolism. Similar thermoregulatory strategies have been observed in nocturnal rodents, where maintaining lower body temperatures during activity at night prevents overheating (Gordon 1994, 2012). In ectotherms, a comparable mechanism may reduce the risk of overheating during periods of high activity. In addition, selecting higher body temperatures during the light period may also facilitate digestive processes during the inactive period. Post‐prandial thermophily has been reported in several reptile species (e.g., Sievert et al. 2005). In the nocturnal gecko *Amalosia lesueurii*, however, although individuals generally select higher body temperatures after fed than after fasted, both fed and fasted individuals consistently select higher temperatures during the light period than during the dark period (Dayananda and Webb 2020). This pattern indicates that the relationship between digestive state and diel thermal preference is not straightforward. Further field studies are needed to determine the adaptive significance of light‐dependent thermoregulation in *P. picta* within its natural habitat.

### 4.2 Temperature Acclimation and Changes in Physical Performance

Although T_sel_ did not differ significantly between acclimation temperatures, CT_min_ and CT_max_ were significantly higher in individuals acclimated to 30°C compared with those acclimated to 25°C. Additionally, sprint speed was higher in 25°C‐acclimated individuals at lower temperatures, whereas 30°C‐acclimated individuals performed better at higher temperatures. These findings indicate that *P. picta* can plastically adjust its physiological functions to accommodate environmental temperatures. However, no significant difference in thermal breadth was observed between the acclimation groups, suggesting a trade‐off: increased high‐temperature tolerance in high temperature–acclimated individuals comes at the cost of reduced tolerance to low temperature. A similar pattern was observed in *Anolis apletophallus* , where long‐term (four‐week) temperature acclimation altered critical temperatures but not T_sel_ (Rosso et al. 2024). Alternatively, one‐week thermal acclimation in *Anolis carolinensis* and *Anolis sagrei* resulted in species‐specific shifts in T_sel_ (Ryan and Gunderson 2021). These differences suggest that thermoregulatory plasticity varies across species and environments, warranting further investigation into the underlying mechanisms. In our CT_min_ and CT_max_ assays, we attempted to approximate a body temperature change of 1°C per minute and took care to avoid excessively rapid shifts in temperature. However, the rate of thermal change could not be controlled perfectly, and therefore we cannot fully exclude the possibility that abrupt temperature fluctuations may have induced physiological stress or shock in some individuals. This limitation should be considered when interpreting the thermal tolerance estimates. With respect to the sprint speed trials, our procedure, which involved briefly exposing individuals to the test temperature before measurement, follows several previous studies employing similar approaches (e.g., Cameron et al. 2018; Cecchetto et al. 2020). Moreover, at least one study comparing acclimation temperatures likewise transferred individuals directly from their acclimation temperature to the test temperature without first fully equilibrating them at a common intermediate temperature (Hodgson and Schwanz 2024). Nevertheless, in our experiment, individuals were moved directly from their acclimation temperature to the test temperature, and the magnitude of this temperature change necessarily differed between acclimation groups. Such differences may have influenced sprint performance and could have contributed to the patterns observed.

The estimated TPC indicated that both P_max_ and T_opt_ shifted upward in 30°C‐acclimated individuals. These results demonstrate that long‐term temperature acclimation not only shifts the temperature range at which individuals are active but also alters physical performance itself. Extending the ‘hotter‐is‐better’ hypothesis, which proposes that populations adapted to higher temperatures achieve greater peak performance owing to increased biochemical efficiency (Angilletta Jr et al. 2010; Logan and Cox 2020), our results suggest that a plastic increase in biochemical efficiency may occur in response to long‐term thermal exposure.

### 4.3 Expression‐Level Responses to Short‐Term Heat Stress and Long‐Term Thermal Acclimation

PCA of gene expression levels showed that Short 30 and Short 37 formed distinct clusters, whereas Long 30 was not distinguishable from Long 25. The clear separation of Short 37 is consistent with the acute exposure to 37°C representing a stressful condition, as suggested by the marked decline in locomotor performance observed near this temperature, and may reflect a characteristic transcriptional response to such short‐term thermal stress. In contrast, long‐term exposure to 30°C was associated at the phenotypic level with a shift in the functional thermal range and an increase in maximal sprint performance, and the absence of strong transcriptional divergence between Long 25 and Long 30 may indicate that initial stress‐related responses became attenuated over prolonged exposure as individuals transitioned to a more stable physiological state compatible with enhanced performance. Notably, individual age and prior environmental experiences were unknown in the present study, and these factors could have influenced sample variability in the PCA.

Among all treatments, Short 37 induced the highest number of DEGs in the brain and liver. This aligns with previous findings that larger temperature fluctuations result in more extensive transcriptional changes (Logan and Cox 2020). GO enrichment analysis of Short 37 DEGs revealed that ‘translation’, ‘gene expression’, ‘mRNA metabolic process’, and ‘protein metabolic process’ were among the most significantly enriched terms in both tissues. ‘Translation’ is frequently reported as a dominant function among temperature‐responsive DEGs (Porcelli et al. 2015), and other functions detected in the brain and liver, such as protein folding and ubiquitination (Mathew and Morimoto 1998), cell death (Samali et al. 1999) and circadian rhythm regulation (Akashi et al. 2016; Chang et al. 2022), have been associated with heat stress responses. Additionally, ‘cellular response to oxidative stress’ (Belhadj Slimen et al. 2014), enriched in the brain, as well as ‘lipid homeostasis’ and ‘lipid storage’ (Porcelli et al. 2015; Yang et al. 2010), detected in the liver, are also known to be involved in these responses.

In contrast to Short 37, significant GO enrichment in Long 30 was observed only in the liver, where immune‐related functions were highly concentrated among the top‐ranking terms. Environmental temperature and seasonal fluctuations are known to influence immune function in ectotherms (Ding et al. 2024; Dittmar et al. 2014; Huyghe et al. 2010; Madelaire et al. 2021). Temperature plays a crucial role in shaping their immune capacity (Rakus et al. 2017; Wojda 2017), and it can also affect the energetic costs associated with mounting and maintaining immune responses (Catalán et al. 2012). In addition, interferon‐mediated antiviral defences exhibit temperature‐dependent variation (Foxman et al. 2015), which is particularly relevant here because many of the enriched functional categories in our study were associated with antiviral pathways, including responses to viral cues, type I interferon signalling and the regulation of RIG‐I and MDA5 related processes, as well as with metabolic pathways that support these immune functions. Although the direction and overall consequences of these immune adjustments remain unclear at this stage, long‐term temperature acclimation may nevertheless reconfigure immune function to align with the prevailing thermal environment.

In addition to the DEG‐level responses described above, the co‐expression patterns provide further insight into how transcriptional regulation differs between acute and prolonged warm exposure. Consistent with our hypothesis, a subset of the gene networks strongly induced under acute heat stress was embedded within the liver green module, whose hub genes include heat‐shock proteins and co‐chaperones (*HSP90AB1*, *DNAJA4*, *HSPA4L*, *DNAJB6*), transcriptional regulators (*YY1*, *JUND*, *NR4A2*) and metabolic enzymes (*GCLC*, *ACLY*, *ASNS*). These genes showed clear positive associations under short‐term heat exposure, whereas under long‐term acclimation the same module tended to exhibit a moderate negative association, although this trend was not statistically significant. The enriched functional categories, such as protein refolding, unfolded‐protein responses, transcriptional regulation and diverse metabolic processes, suggest that prolonged warm exposure may dampen not only heat‐stress responses but also other components of the acute, energetically demanding reaction to sudden temperature elevation. Such attenuation could facilitate phenotypic optimisation by reducing the costs of sustained stress‐related transcriptional activity and enabling metabolic processes to operate more efficiently at the acclimation temperature. In contrast, the overlapping module in the brain (the yellow module) did not show comparable indications of downregulation under long‐term acclimation, suggesting that acclimation‐driven adjustments to transcriptional regulation may follow distinct trajectories across tissues and reflect their different physiological roles and thermal sensitivities.

### 4.4 Functions of DSGs Identified Under Short‐ and Long‐Term Temperature Exposure Treatments

The DSGs identified in response to temperature changes largely differed from the DEGs, indicating that transcript abundance and alternative splicing function as independent regulatory mechanisms. GO enrichment analysis revealed that splicing‐related terms were among the most prominent across all treatment groups where enrichment was detected. Similar findings have been reported in catfish, where temperature fluctuations induced splicing changes in splicing‐related genes (Tan et al. 2019).

In the brain, DSGs were enriched for functions related to neural communication, with ‘synaptic vesicle budding from presynaptic endocytic zone membrane’ enriched in Long 30 and ‘neuron development’ and ‘dendrite morphogenesis’ in Short 37. In the liver, Long 30 induced changes in genes related to tyrosine metabolism. Tyrosine serves as a precursor for catecholamines (Daubner et al. 2011) and melanin in the skin (Riley 1997) and is primarily synthesised in the liver (Hsieh and Berry 1979). Catecholamines act as stress hormones that regulate heart rate, metabolism and heat shock protein production in response to heat stress (Alfonso et al. 2021). In fish and rodents, long‐term temperature acclimation has been shown to modulate the catecholaminergic system (Alfonso et al. 2021; Nakagawa and Ishiwata 2021). Long‐term acclimation to 30°C was associated at the phenotypic level with both an upward shift in the functional thermal range and an increase in maximal performance. Because catecholamines derived from tyrosine regulate metabolic rate, cardiovascular function and muscle activity, temperature‐induced modulation of tyrosine metabolism may influence the physiological capacity needed to sustain higher performance at elevated temperatures. In particular, adjustments in catecholaminergic pathways could alter energy mobilisation, oxygen delivery or neuromuscular responsiveness in ways that are consistent with the enhanced sprint capacity observed in warm‐acclimated individuals, although the present study does not directly test these mechanisms.

### 4.5 Relationship Between Chromatin Accessibility and Gene Transcription

More than half of the genomic regions with temperature‐induced chromatin accessibility changes were not located near genes, and only a small fraction of DPGs overlapped with the DEGs or DSGs. This result was unexpected, given our initial hypothesis that temperature‐responsive gene expression and splicing would be closely coupled to local chromatin remodelling. This suggests that chromatin accessibility changes do not necessarily drive nearby gene expression changes. However, over 85% of DEGs and DSGs had at least one ATAC‐seq peak in their vicinity in either the control or treatment groups, suggesting that these genes remained in a generally accessible state regardless of temperature fluctuations. A similar pattern was observed in rats, where more than half of temperature‐responsive DPGs did not overlap with DEGs (Dou et al. 2022), indicating that large‐scale fluctuations in chromatin accessibility may not be the primary regulator of temperature‐responsive transcription in neighbouring genes. These results further suggest that many temperature‐induced changes in gene expression and splicing may be mediated by mechanisms other than broad chromatin remodelling, such as cascades of temperature‐responsive transcription factors or post‐transcriptional regulation by RNA‐binding and splicing factors. Clarifying the relative contributions of these mechanisms will be essential for understanding temperature‐induced transcriptional and splicing plasticity. Nevertheless, some DEGs and DSGs exhibited significant chromatin accessibility changes in their vicinity. For instance, *HSPA8* and *DNAJA4*, identified in Short 37, encode heat shock proteins that protect cells from stressors, including heat (Hu et al. 2022). *HSPA8* was also identified as a hub temperature‐responsive gene in the lizard *Eremias argus* (Chang et al. 2022). Notably, *DNAJA4* was a hub gene associated with temperature treatments in our study, and it has also been reported to show temperature‐dependent expression changes across three anole lizard species (Akashi et al. 2016). These findings suggest that acute temperature responses may be dynamically regulated by chromatin remodelling, particularly for genes essential for heat stress protection.

### 4.6 Transcription Factors With Predicted Changes in Occupancy Under Short‐ and Long‐Term Temperature Treatments

In the liver, most transcription factors that exhibited changes in predicted occupancy across multiple temperature conditions showed opposing patterns between short‐ and long‐term treatments. This suggests that, although acute responses to rapid temperature changes are activated initially, prolonged exposure to high temperatures suppresses these pathways. Acute thermal stress responses typically involve the rapid induction of processes related to proteostasis, cellular damage repair and metabolic reorganisation, and these responses are energetically expensive and difficult to sustain over extended periods (Feder and Hofmann 1999). Maintaining such high‐cost pathways chronically can divert metabolic resources from other physiological functions. Accordingly, their suppression during long‐term warm exposure may reflect a shift toward more economical and sustainable modes of physiological regulation.

Among these transcription factors, the C/EBP family plays a key role in regulating cell differentiation, nutrient metabolism, adipogenesis and immune responses, with its expression fluctuating in response to acute inflammatory stimuli (Ko et al. 2015; Peirce et al. 2014; Poli 1998). Several studies have reported temperature‐induced changes in C/EBP gene expression, although the direction of change has varied across these studies (Bloomer et al. 2014; Buckley 2011; Dou et al. 2022; Sleadd and Buckley 2013). In the present study, CEBPB and CEBPD showed increased expression under Short 37, whereas CEBPA and CEBPG exhibited decreased expression under Long 30, indicating that different members of the C/EBP family respond in distinct ways depending on the duration and intensity of thermal exposure. A comparable directional shift was also suggested at the network level: the WGCNA results showed that a portion of the gene networks strongly induced under acute heat stress tended to exhibit reduced activity under long‐term warm acclimation, supporting our hypothesis that prolonged exposure suppresses acute, strong heat‐stress responses rather than maintaining them. Such coordinated adjustments across transcription factor networks may facilitate the transition from an acute stress response to a stabilised transcriptional state characteristic of long‐term temperature acclimation.

Several DEGs with changes in the footprint scores of C/EBP family transcription factor binding sites in their promoter regions were identified. These included genes involved in lipid metabolism, namely *FMO5*, *APOVLDLII*, *PLIN2*, *HMGCS1*, *ABHD4* and *DUSP16* (Alphonse and Jones 2016; Q. Chen et al. 2021; Gonzalez Malagon et al. 2015; Price 2017; Seramur et al. 2023; Y.‐K. Wu et al. 2020), as well as genes associated with immune responses, including *PDE12*, *CLTC*, *IFNAR2*, *VDR* and *MINA* (Brodsky 2012; Oh et al. 2019; Stetson and Medzhitov 2006; Wood et al. 2015; S. Wu and Sun 2011). These genes represent strong candidates for mediating temperature‐responsive physiological changes under C/EBP family regulation. However, as transcription factors with similar structures often bind to overlapping sequences, it remains unclear which specific C/EBP family members were directly responsible for these changes. Further studies are needed to clarify their precise regulatory roles.

## 5 Conclusion

This study examined the temperature response profile of *P. picta* and demonstrated that long‐term exposure to high temperatures plastically shifts the range of body temperatures at which individuals remain active. Molecular analyses revealed distinct transcriptional and regulatory responses depending on the duration of thermal exposure: acute temperature changes primarily activated known heat stress pathways, whereas long‐term acclimation led to shifts in immune function. Additionally, in the liver, some transcription factors exhibited opposing changes in the predicted occupancy of transcription factors between short‐ and long‐term conditions, suggesting that these factors may contribute to the transition from immediate thermal stress responses to acclimatory adjustments. These findings highlight how the duration of temperature exposure drives distinct molecular responses, providing a foundation for understanding the chronological process of thermal acclimation. However, the direct physiological mechanisms linking these molecular changes to shifts in physical performance remain unclear. Future studies focusing on phenotypic changes in physiological functions will be crucial in bridging the gap between molecular‐level changes and whole‐body thermal acclimation.

## Author Contributions

Conceptualisation: F.S. and M.K.; Methodology: F.S., S.K., T.M. and M.K.; Investigation: F.S., F.R., M.K.; Visualisation: F.S.; writing – original draft: F.S.; writing – review and editing: F.S., S.K., F.R., T.M. and M.K.; Formal analysis: F.S.; Software: F.S.; Resources: F.S., T.M. and M.K.; Data curation: F.S.; Funding acquisition: F.S. and M.K.; Supervision: M.K.; Project administration: M.K.

## Funding

This work was supported by Human Frontier Science Program, RGP0030/2020 and Japan Society for the Promotion of Science, 16H05767, 19KK0184.

## Disclosure

Benefit‐Sharing Statement: A research collaboration was developed with scientists from the countries allowing use of genetic samples (Permits from the General Management of Environmental Governance, Madagascar, no. 176/23/MEDD/SG/DGGE/DAPRNE/SCBE.Re); all collaborators are included as co‐authors; the results of research have been shared with the provider communities and the broader scientific community; and the research addresses a priority concern, in this case the ecology, evolution and conservation of organisms being studied. More broadly, our group is committed to international scientific partnerships, as well as institutional capacity building.

## Conflicts of Interest

The authors declare no conflicts of interest.

## Supporting information


**Data S1:** mec70245‐sup‐0001‐FiguresS1‐S6.pdf.

**Data S2:** mec70245‐sup‐0002‐TablesS1‐S4.xlsx.

**Data S3:** mec70245‐sup‐0003‐TablesS5‐S18.xlsx.

## Data Availability

Raw sequence reads are deposited in the SRA (BioProject PRJDB20333). The measurement data from behavioural experiments, the codes used for the analyses conducted in this study, the results of read mapping, expression quantification and alternative splicing event detection using RNA‐seq data, as well as the results of read mapping, peak calling and footprint analysis using ATAC‐seq data, are available on Dryad (https://doi.org/10.5061/dryad.pvmcvdnwm).
