# Supplementary material for: Molecular Responses to Temperature Changes Across Timescales in the Madagascar Ground Gecko (Paroedura picta)
Source: Mol Ecol. 2026 Jan 17;35(2):e70245. doi: 10.1111/mec.70245 (PMC12811822; doi:10.1111/mec.70245)
Supplement: Supplementary file 1 — Data S1: mec70245‐sup‐0001‐FiguresS1‐S6.pdf. [file MEC-35-e70245-s002.pdf]

MOLECULAR ECOLOGY

Supplemental Information for:

Molecular Responses to Temperature Changes Across Timescales in the Madagascar Ground Gecko (*Paroedura picta*)

Fuku Sakamoto, Shunsuke Kanamori, Félix Rakotondraparany, Takashi Makino, Masakado Kawata

Table of Contents:

**Figure S1**	Page 2
**Figure S2**	Page 3
**Figure S3**	Page 4
**Figure S4**	Page 5
**Figure S5**	Page 6
**Figure S6**	Page 7

MOLECULAR ECOLOGY

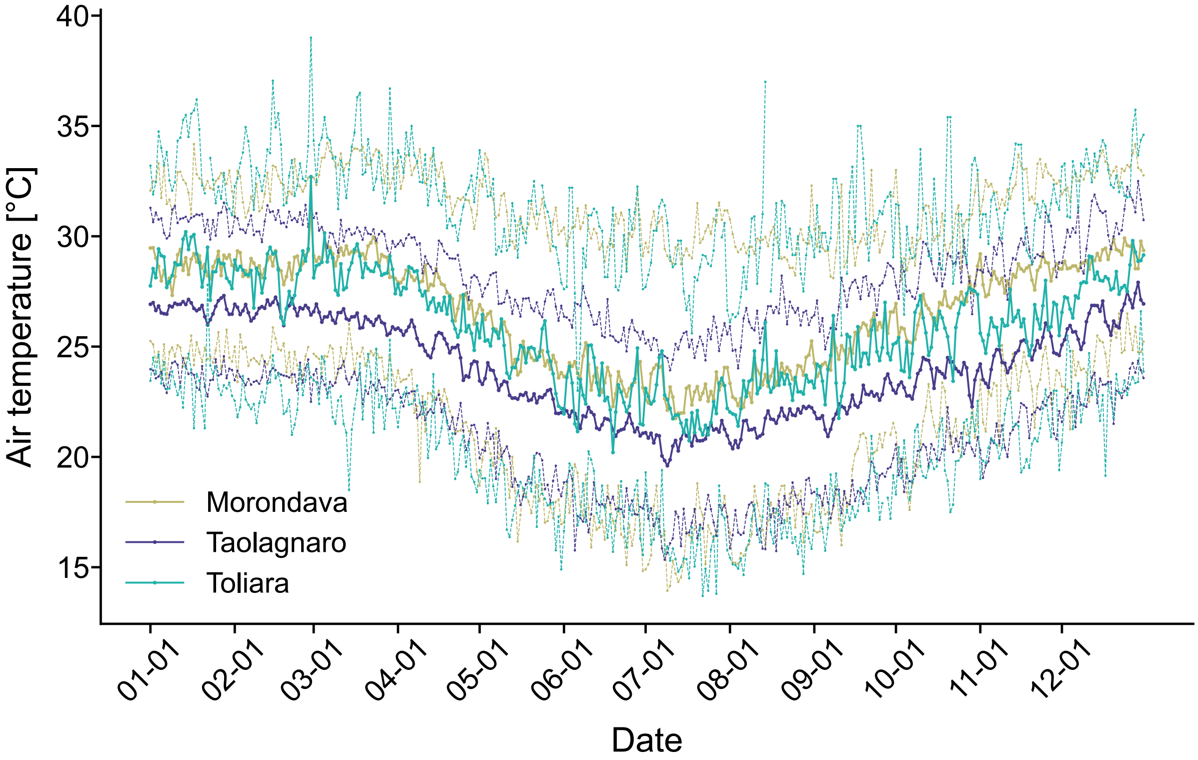

Figure S1. Annual temperature trends in three cities near the habitat of *P. picta*. Daily mean temperature (solid line), maximum temperature (upper dashed line), and minimum temperature (lower dashed line) were calculated as averages using all available daily data from 2010 to 2020. Yellow: Morondava; navy: Taolagnaro; cyan: Toliara.

MOLECULAR ECOLOGY

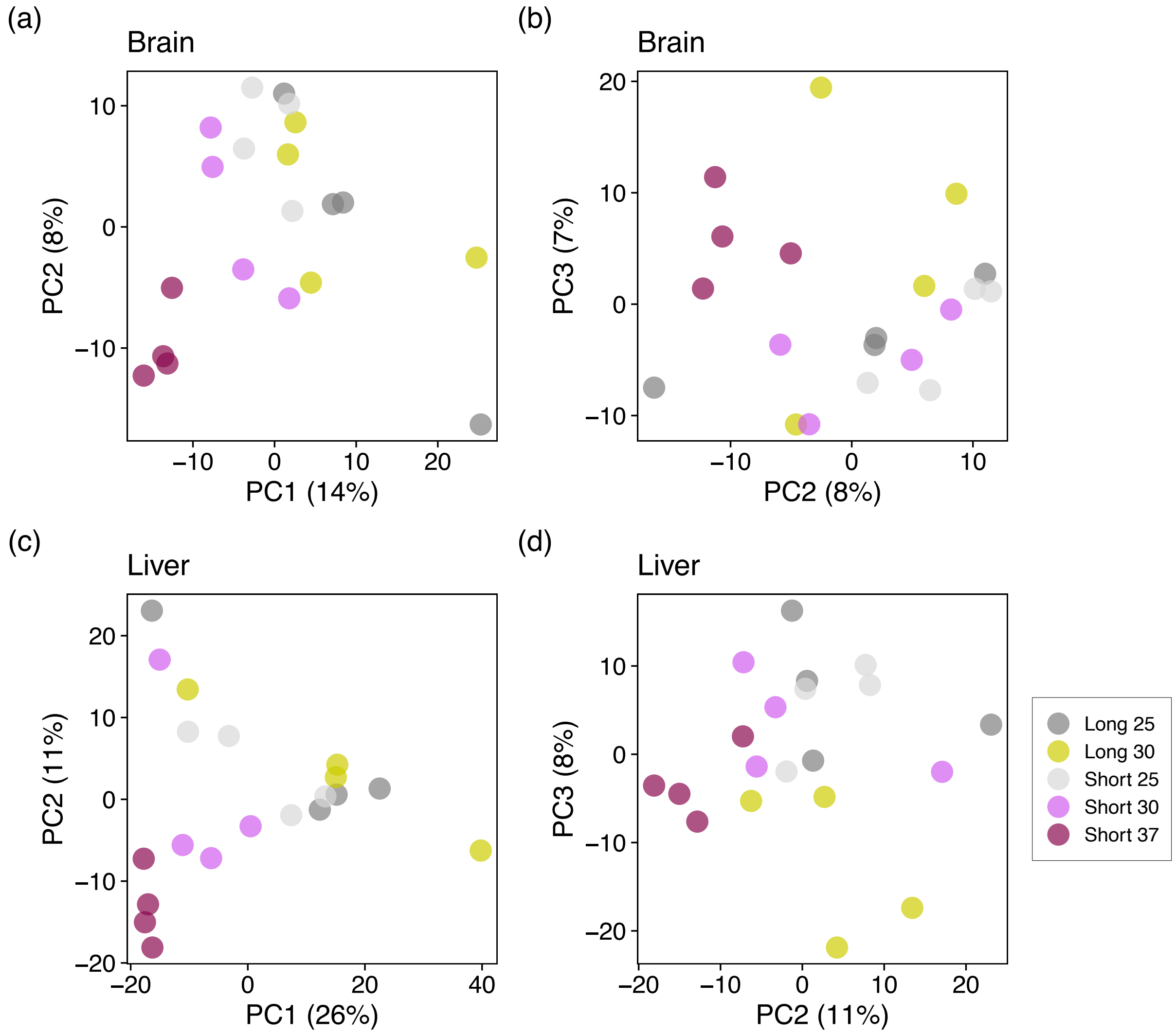

Figure S2. Principal component analysis of gene expression in the brain (a, b) and liver (c, d). Each point represents the gene expression profile of an individual sample. Numbers in parentheses indicate the proportion of variance explained by the principal components. PC1, PC2, and PC3: first, second, and third principal components, respectively.

MOLECULAR ECOLOGY

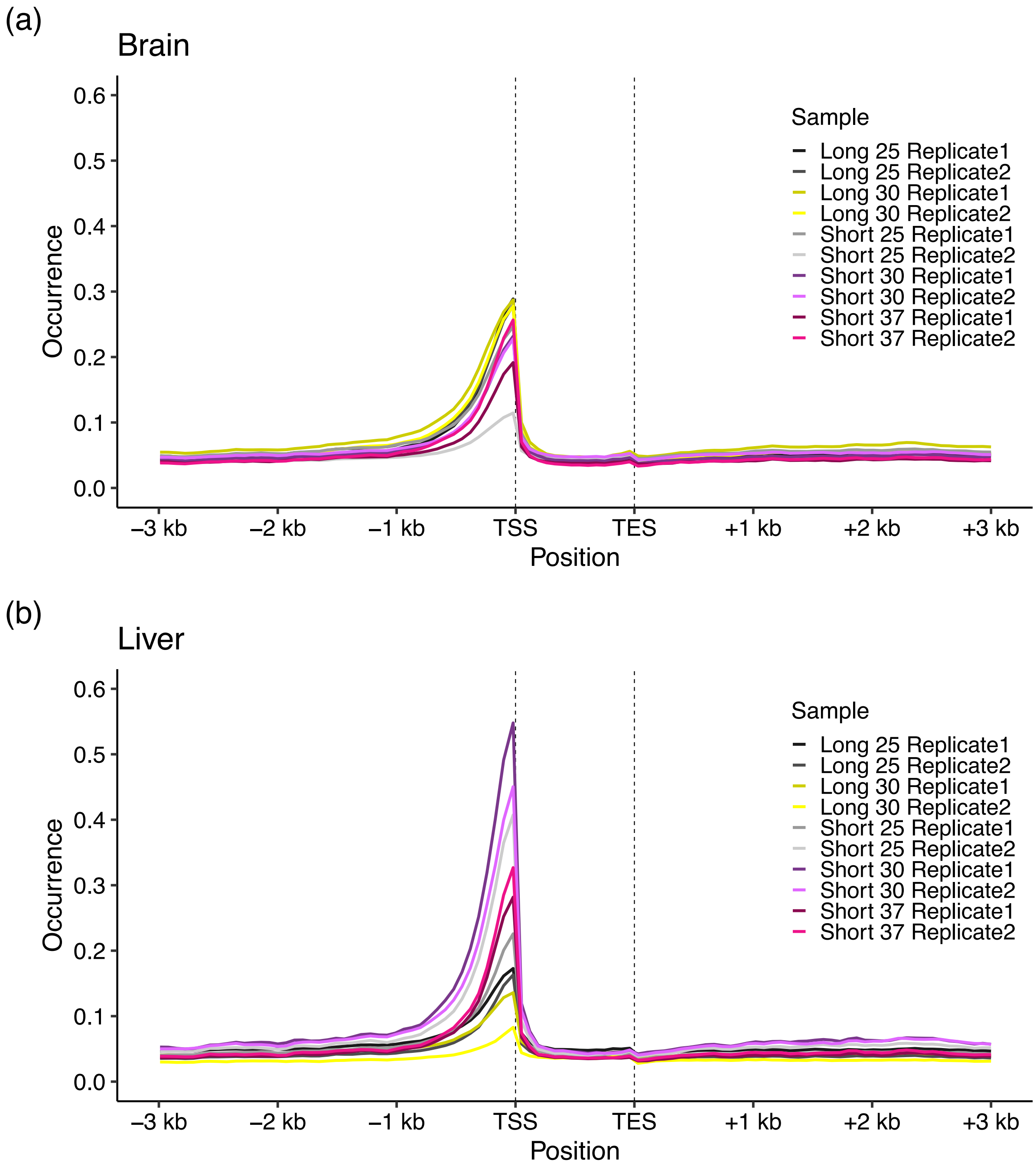

Figure S3. Distribution of ATAC-seq reads around gene regions in the brain (a) and liver (b). Gene sizes are compressed within the displayed range. TSS, transcription start sites; TES, transcription end sites.

MOLECULAR ECOLOGY

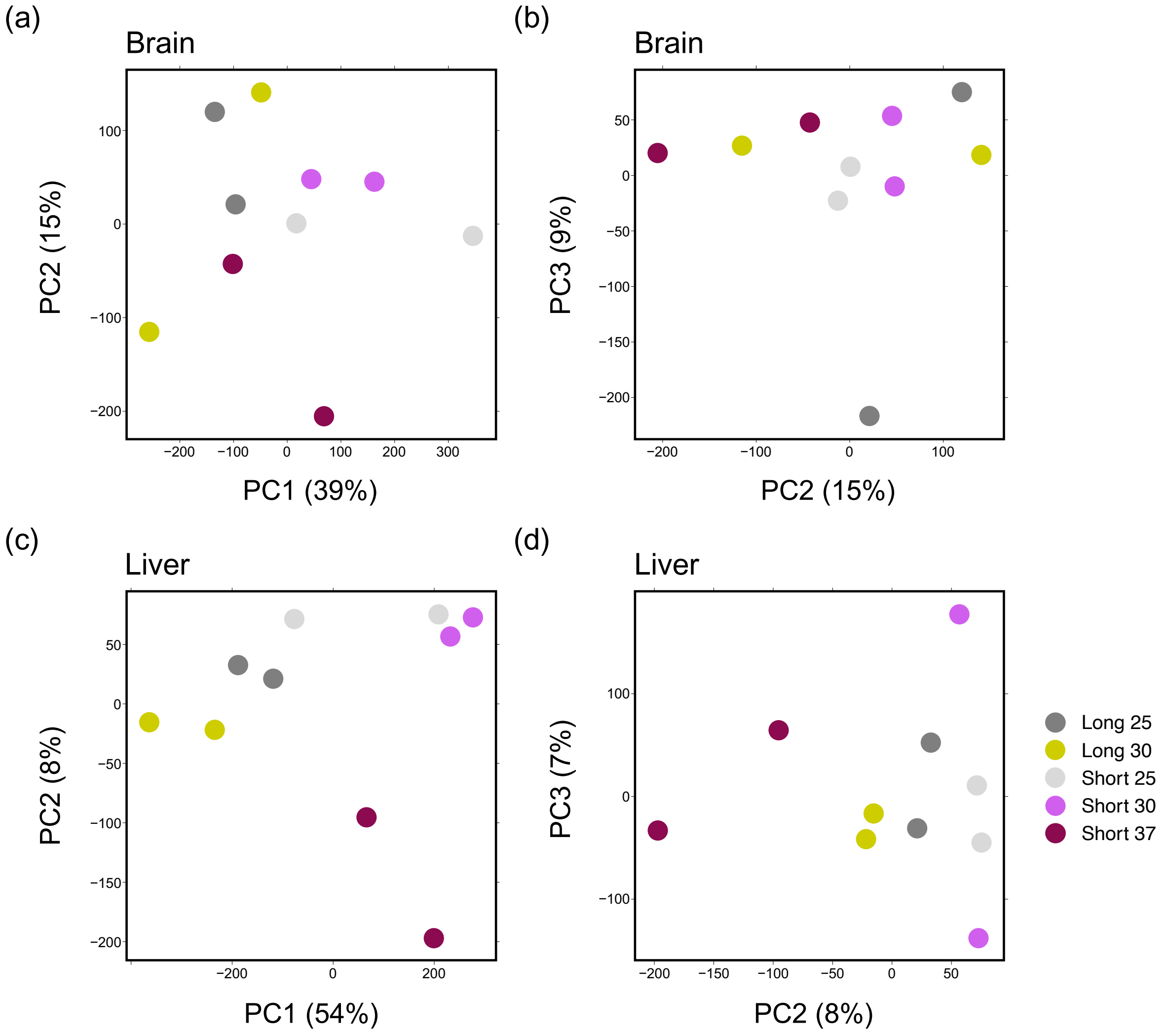

Figure S4. Principal component analysis of chromatin accessibility signals in the brain (a, b) and liver (c, d). Each point represents the accessibility profile of an individual sample. Numbers in parentheses indicate the proportion of variance explained by the principal components. PC1, PC2, and PC3: first, second, and third principal components, respectively.

MOLECULAR ECOLOGY

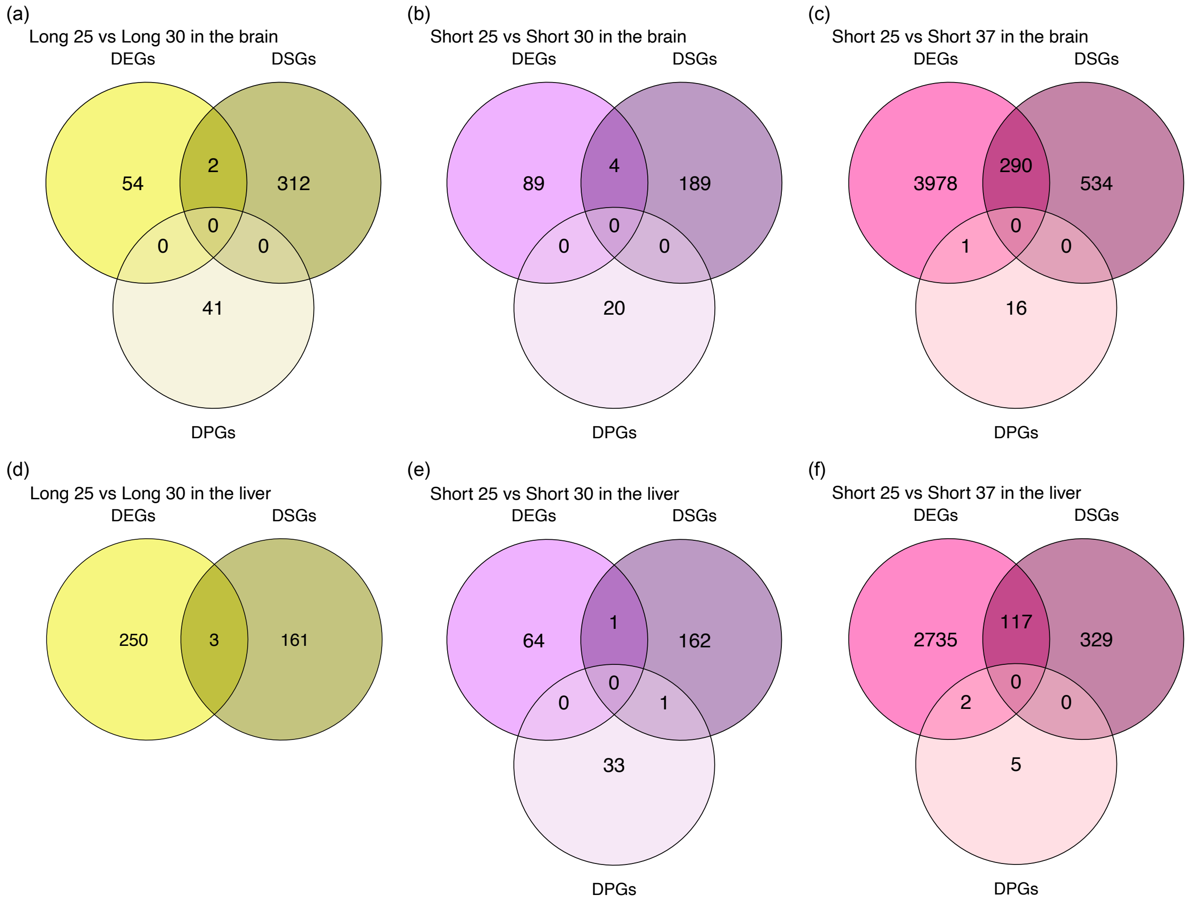

Figure S5. Comparison of genes with altered expression, splicing, and nearby chromatin accessibility in response to temperature changes in the brain (a–c) and liver (d–f). Treatment groups (Long 30: exposure to 30°C for >50 days; Short 30 and Short 37: exposure to 30°C or 37°C for 4 hours) were compared with the control group (25°C) over the same duration (Long 25: exposure to 25°C for >50 days; Short 25: exposure to 25°C for 4 hours). DEGs, differentially expressed genes; DSGs, differentially spliced genes; DPGs, differential peak-associated genes.

MOLECULAR ECOLOGY

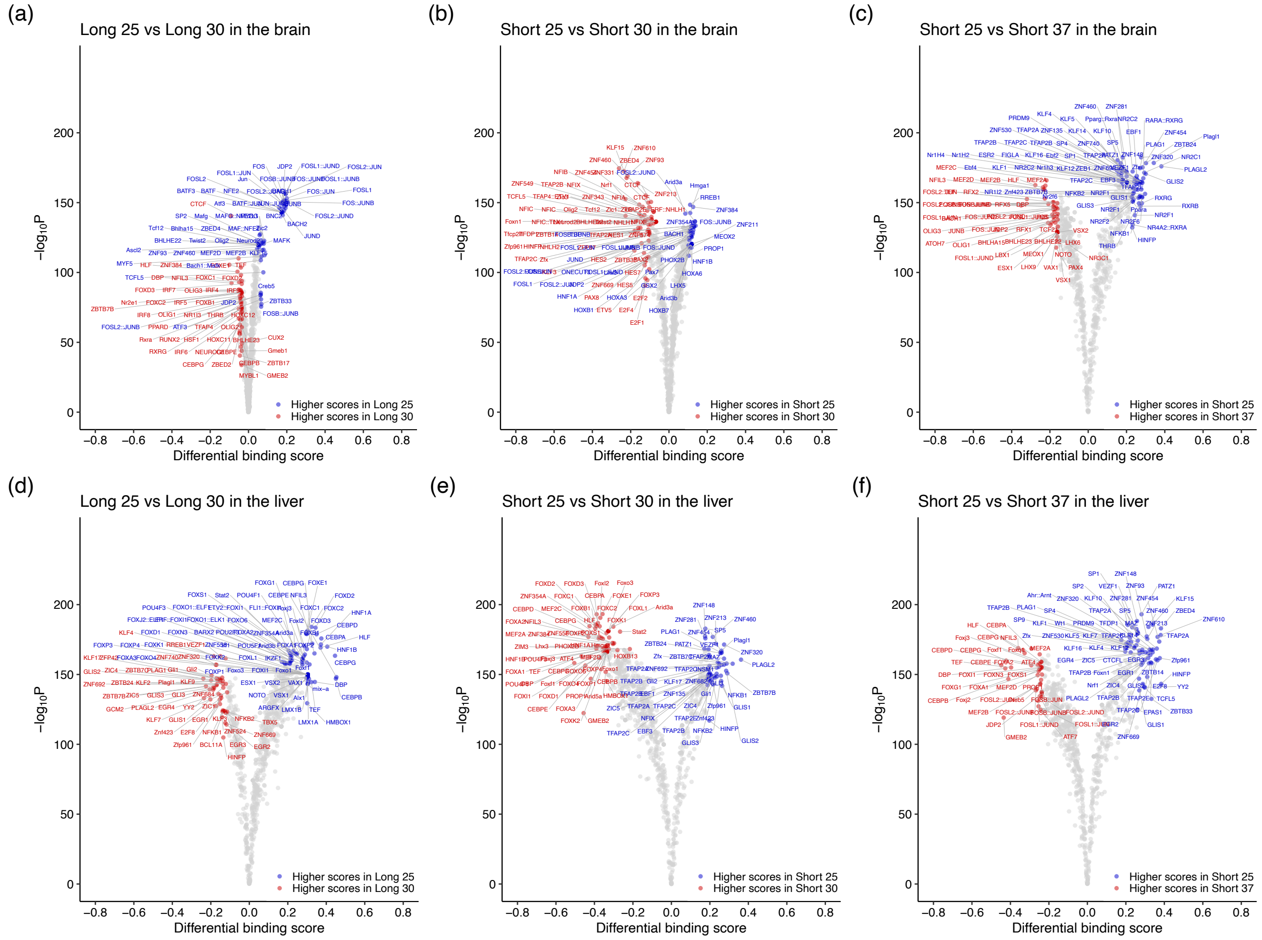

Figure S6. Volcano plots showing predicted changes in transcription factor occupancy between temperature conditions in the brain (a–c) and liver (d–f). Treatment groups (Long 30: exposure to 30°C for >50 days; Short 30 and Short 37: exposure to 30°C or 37°C for 4 hours) were compared with the control group (25°C) over the same duration (Long 25: exposure to 25°C for >50 days; Short 25: exposure to 25°C for 4 hours).
